# High-order social interactions in groups of mice

**DOI:** 10.7554/eLife.00759

**Published:** 2013-09-03

**Authors:** Yair Shemesh, Yehezkel Sztainberg, Oren Forkosh, Tamar Shlapobersky, Alon Chen, Elad Schneidman

**Affiliations:** 1 Department of Neurobiology, Weizmann Institute of Science, Rehovot, Israel; 2 Max Planck Institute of Psychiatry, Munich, Germany; University of Chicago, United States

**Keywords:** group behavior, social interaction maps, maximum entropy models, tracking individuals in groups, environmental complexity, pairwise and high-order correlations, Mouse

## Abstract

Social behavior in mammals is often studied in pairs under artificial conditions, yet groups may rely on more complicated social structures. Here, we use a novel system for tracking multiple animals in a rich environment to characterize the nature of group behavior and interactions, and show strongly correlated group behavior in mice. We have found that the minimal models that rely only on individual traits and pairwise correlations between animals are not enough to capture group behavior, but that models that include third-order interactions give a very accurate description of the group. These models allow us to infer social interaction maps for individual groups. Using this approach, we show that environmental complexity during adolescence affects the collective group behavior of adult mice, in particular altering the role of high-order structure. Our results provide new experimental and mathematical frameworks for studying group behavior and social interactions.

**DOI:** http://dx.doi.org/10.7554/eLife.00759.001

## eLife digest

All animals need to interact with others of the same species, even if it is only to mate. To date, social behavior has been studied mainly at two extremes: detailed observation of pairs; and studies of the collective behavior of large groups, such as flocks of birds. However, to gain an understanding of social behavior in mammals will require an approach that falls between these two extremes. It will be necessary to study animals in larger groups, rather than in pairs, but also to track individuals rather than looking at the activity of the group as a whole.

Now, Shemesh et al. have developed a system that can track the behavior of each of four mice with high spatial and temporal resolution as they move around freely in an arena containing ramps, nest boxes, and barriers. Because mice are largely nocturnal, Shemesh et al. dyed the animals’ fur with compounds that produced different coloured fluorescence under ultraviolet light, and then employed an automated system to accurately track each mouse during 12 hr of darkness, over a number of days.

Using these data it was possible to estimate the extent to which the behavior of the group is determined by the characteristics of individual mice and how much is determined by interactions between animals. Models based solely on the behavior of individuals could not accurately describe the behavior of the group. Surprisingly, neither could models that focused on interactions between pairs of mice. Only models that included interactions between three mice gave a good approximation of the observed behavior. This shows that, even in a small group, social behavior is determined by relatively complex interactions.

Shemesh et al. also found that the behavior of the mice depended on the environment in which they had been raised. Animals that had lived in larger groups and in more interesting enclosures were influenced more by pairwise interactions, and less by three-way interactions, than mice that had been raised in a standard laboratory environment. This suggests that being raised in a complex environment strengthens mouse ‘individuality’. The approach developed by Shemesh et al. could be extended to study larger groups of animals and could also be used to examine the interplay between genes, environment and other factors in shaping social interactions.

**DOI:** http://dx.doi.org/10.7554/eLife.00759.002

## Introduction

Understanding the nature and impact of the interactions that underlie the behavior of groups of organisms is a central question, shared across biology, physics, psychology, and mathematics. The coherence of ‘collective behavior’ patterns of large groups of animals, such as insect swarms (Buhl et al., 2006; Simpson et al., 2006) fish schools (Sumpter et al., 2008; Katz et al., 2011), bird flocks (Cavagna et al., 2010; Nagy et al., 2010), and human crowds (Song et al., 2010; Gallup et al., 2012), presents us with fundamental questions relating to distributed information processing, computation, and learning. The adequacy of different mathematical models of the interactions between animals in describing the behavior of large groups has therefore been of great interest (Winfree, 1967; Vicsek et al., 1995; Couzin et al., 2002; Lathe, 2004; Couzin et al., 2005; Ben-Jacob, 2009; Cavagna et al., 2010; Lukeman et al., 2010; Nagy et al., 2010; Bialek et al., 2012).

Smaller groups of animals present an interesting and sometimes more difficult scenario, where individual traits may play an important role in shaping the group behavior (Lathe, 2004). This may be especially true in mammals, where both individual behavior and interactions are often assumed to be more complex. It has therefore been common to study ‘social behavior’ in small groups and explore the interplay of individual and group relations in decision-making (Couzin et al., 2011), information transfer (Leadbeater and Chittka, 2007), learning (Couzin et al., 2005), and more. Yet, much of our understanding of social behavior has come from studies of just pairs of animals under artificial settings (Insel and Fernald, 2004; Langford et al., 2006; Moy et al., 2008; Branson et al., 2009; Dankert et al., 2009; Blumstein et al., 2010; Silverman et al., 2010; Ben-Ami Bartal et al., 2011; de Chaumont et al., 2012). It is not clear, however, what the detailed analysis of social interaction at the level of a single pair implies for larger groups. In particular, new features may emerge that characterize the group as a whole that cannot be inferred from the study of individuals or pairs (Cavagna et al., 2010).

To study the nature of interactions underlying social behavior in a group, we used a novel automatic system for tracking individuals in small groups of mice over long periods of time, in an environment that is ethologically relevant. Systems for tracking individual animals in simple and enriched environments have been used in recent years to characterize individual behavior (Branson et al., 2009; Green et al., 2012; Freund et al., 2013), and even to relate individual behavior to neurogenesis (Freund et al., 2013). We focused here on the nature of group behavior, and in particular how group behavior is the result of individuality, pairwise, and potentially higher-order interactions between the animals. We then used a maximum entropy-based modeling framework (Jaynes, 1957; Schneidman et al., 2003, 2006) to quantify the nature of correlated group behavior and map the social interactions between individuals. Finally, we compared the joint activity patterns and social contacts of mice subjected to environmental manipulations.

## Results

**Figure 1.**
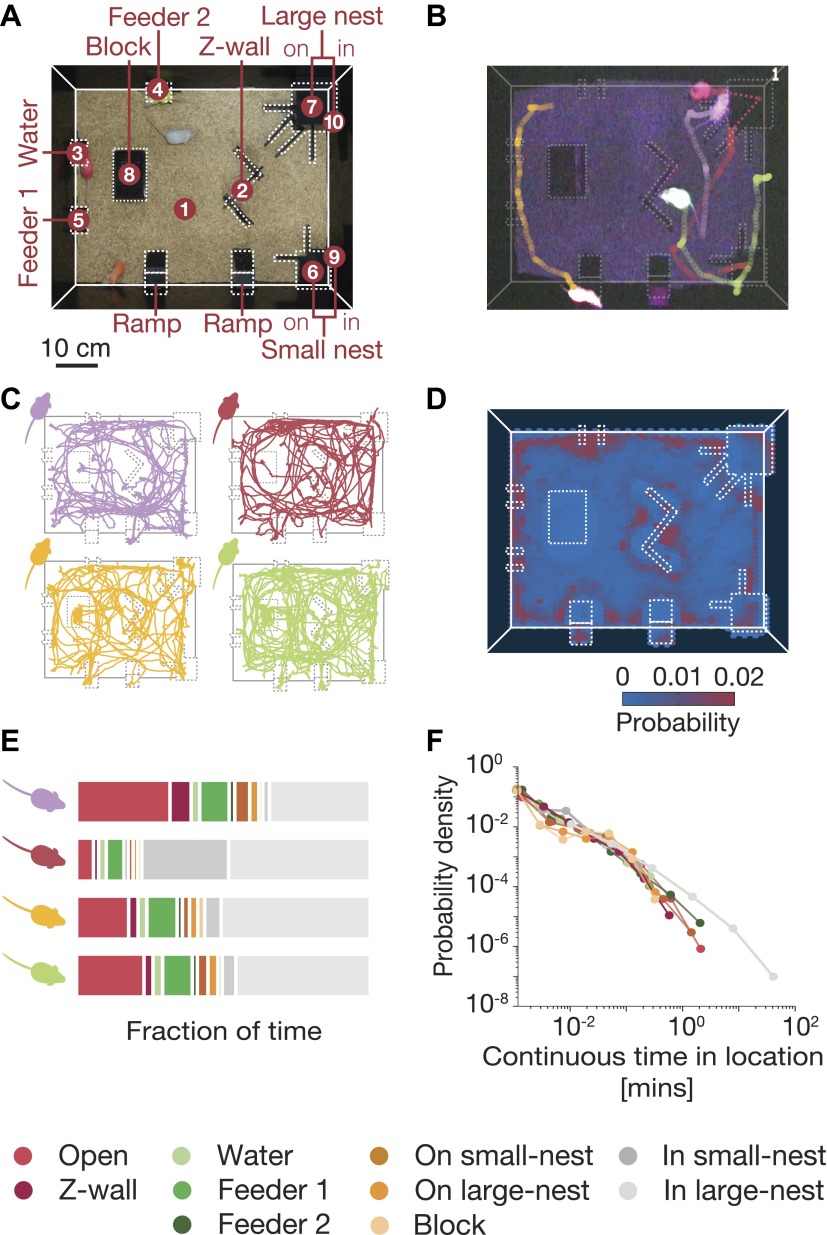
Simultaneous tracking of individual mouse behavior in the dark. (**A**) Top view of the arena showing the 10 regions of interest: (1) open field, (2) Z wall, (3) water, (4 and 5) feeders, (6) on small nest, (7) on large nest, (8) block, (9) in small nest, (10) in large nest. For further details of the arena, see Figure 1—figure supplement 1. (**B**) Video recording and color-based tracking of a group of mice in the dark. (**C**) A 15-min segment of the tracked paths of each of the four mice in a group. (**D**) A ‘heat map’ showing the relative amount of time the mice spent in different parts of the arena. Data shown is from one typical group on the second day of the experiment (red corresponds to highly visited points, and blue to less favorable ones). (**E**) Individual histograms of the time spent in the different regions of the arena (same group as in **D**). See legend at the bottom for the color coding of the regions. (**F**) Distribution of continuous time periods spent by one typical mouse at each region. Most areas show a similar behavior resembling scale free distribution. **DOI:** http://dx.doi.org/10.7554/eLife.00759.003

We analyzed the behavior of 17 groups of mice, each composed of four adult mice in an arena resembling an ethologically relevant context that has an interesting environment for exploration (see ‘Materials and methods’) (Figure 1A, and Figure 1—figure supplement 1). Mice were raised either in a standard laboratory environment (SE mice; eight groups) or an enriched one (CE mice, nine groups; see ‘Materials and methods’) and studied in the arena using a novel automated system for tracking individual and group behavior simultaneously, with high spatial and temporal resolution. To enable accurate tracking of the mice in their nocturnal phase, we stained their fur with fluorescent hair dyes, illuminated the arena with UVA light, and recorded their activity with a sensitive color camera (Figure 1B). The behavior of each group was recorded at 25 frames/s, over 4 consecutive nights, for 12 hr each night (Figure 1C and Video 1; see ‘Materials and methods’).

**Figure 1—figure supplement 1.**
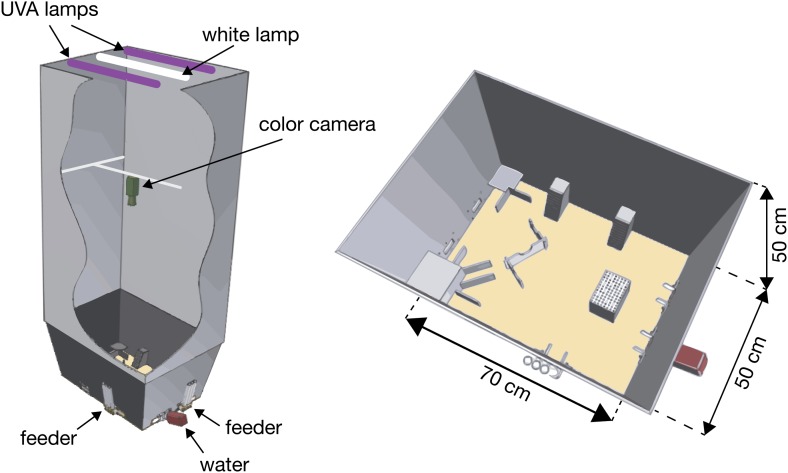
The color-based tracking system. A special arena for automated tracking of individual and group behavior was designed and constructed. The arena consisted of a 70 cm × 50 cm × 50 cm cage that included several objects: a Z-shaped wall, a water dispenser, two feeders, a small nest and a large nest, an elevated block, and two elevated ramps. Two UVA fluorescent lamps were placed 3 m above the arena floor to illuminate the surrounding area during the night with 370–380 nm wavelength light. During the day the arena was lit by one white-light fluorescent lamp (36W). To prevent reflections from objects in the room, a black curtain was drawn from the fluorescent lights down to the arena. A color sensitive camera was placed 1 m above the arena. **DOI:** http://dx.doi.org/10.7554/eLife.00759.004

**Video 1.** Tracking four mice in a semi-natural environment. Each mouse is stained using a different fluorescent hair dye that glows under ‘dark’ light (UVA). The positions of the mice were constantly tracked and the trails are shown in the video. The arena included several objects, which we marked using dashed line in the video. In each frame, the location of all mice is presented in the lower left side of the video by a 4-digit code, which is based on a division of the arena into 10 regions of interest. **DOI:** http://dx.doi.org/10.7554/eLife.00759.005

Mice spent much more time in certain locations in the arena (see ‘heat map’ in Figure 1D). We therefore used a discretized representation of their spatial configurations, based on the ten most visited regions of interest in the arena. We found that individual mice in the group had distinct personal preferences for certain locations (Figure 1E), and the relative amounts of time spent in different places. Despite these individual differences, the amount of time that each mouse spent continuously at each location had a power-law-like behavior (Figure 1F). This power-law distribution was similar for the different mice, when they were each analyzed individually, despite their individual differences on other parameters. It is unclear, however, how one should interpret these individual characteristics of the mice, given that they share the same space. We therefore moved on to consider what portion of the joint configurations can be explained in terms of individual behavior, and how are the mice affected by their peers, that is via social interactions?

### Strongly correlated group behavior among mice

**Figure 2.**
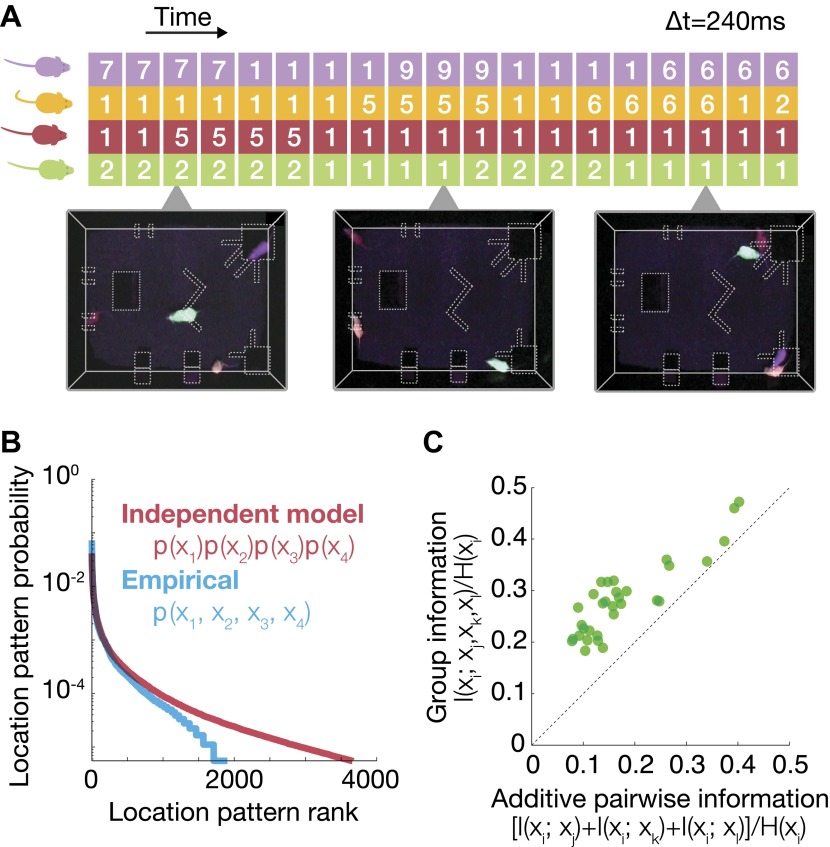
Characterization of group behavior patterns, and signatures of strong group correlations between mice. (**A**) The joint configuration of the mice at each time frame was represented by a 4-dimensional vector, where each dimension denotes the location of a particular mouse in 1 of the 10 regions of interest. (**B**) Comparison between the empirical probability distribution of the observed configurations and a predicted distribution from a model that assumes independence between mice. Configurations were ranked from the most to the least prevalent. (**C**) Fraction of uncertainty about the location of a mouse that can be read from the location of other group members (mutual information about location, divided by location entropy). Every dot shows the fraction of information about the location of mouse i that can be read from the joint location of the other three vs the sum of pairwise information terms between i and each of the other mice. Each of the 32 dots corresponds to 1 mouse (4 mice in 8 groups), and the information that can be read from his group members. The results are for day 2 of the test. **DOI:** http://dx.doi.org/10.7554/eLife.00759.006

To characterize the behavior of the mice as a group, we studied their joint spatial configurations over long periods of time. We defined the ‘state’ of the group at time t, as a vector, (*x*_1_, *x*_2_, *x*_3_, *x*_4_), where *x*_*i*_ denotes the location of mouse i at that time, and *x*_*i*_ = 1,…,10, denote the regions defined in Figure 1. An example of these state vectors as a function of time is shown in Figure 2A, with time bin of Δt = 240 ms (this choice did not affect the results over a wide range of values, see ‘Materials and methods’). We then compared the empirical probability to find the group in a given spatial configuration p_empirical_ (*x*_1_, *x*_2_, *x*_3_, *x*_4_) with the prediction of a model that assumes that the mice choose their locations independently, based on their individual preferences, p_ind_ (*x*_1_, *x*_2_, *x*_3_, *x*_4_) = p(*x*_1_) p(*x*_2_) p(*x*_3_) p(*x*_4_). This difference is exactly the extent to which the group is different from the case of a collection of independent individuals. We found that the two distributions were very distinct, that is the group behavior is very different from what one would expect from studying single mouse properties. In particular, Figure 2B shows the distribution of observed states for a typical group, where out of the 10^4^ possible states (of 4 mice in 10 zones), only approximately 2000 occurred in the experiment, whereas the independent model predicts that approximately 4000 states would typically occur in our experiment. In other words, the correlations between mice contract the space of ‘allowed’ configurations, such that many of them are socially avoided.

To quantify the strength of dependencies between the mice, we first asked how much does knowing the location of one mouse tells us about that of the others. (If the mice were completely individual and ignored one another, then knowing the location of one mouse would tell us nothing about that of the others.)

Since the entropy of the distribution of locations of a mouse, $H\left(x_{i}\right)=-\sum_{x_{i}}\text{p}\left(x_{i}\right)\log_{2}\text{p}\left(x_{i}\right)$ measures how much we do not know about its location, then the dependency between mice can be measured in terms of how much of this uncertainty is reduced by knowing the location of another mouse. This is exactly the mutual information $I\left(x_{i};x_{j}\right)=H\left(x_{i}\right)-H\left(x_{i}|x_{j}\right)$ between the location of one mouse *x*_*i*_ and that of the other mouse *x*_*j*_. To get a normalized measure of the fraction of uncertainty about the location of mouse *i* that can be ‘read’ from mouse *j*, we divided $I\left(x_{i};x_{j}\right)$ by the entropy of location of mouse *i*, *H*(*x*_*i*_). We found that knowing the location of one mouse gives relatively little information about the location of another—typically less than 5% (averaged on all pairs, Figure 5—figure supplement 1). However, knowing the joint locations of three mice gives much more information about that of the fourth one—over all groups and mice $I\left(x_{i};\left\{x_{j},x_{k},x_{l}\right\}\right)$ was, on average, 25% of *H*(*x*_*i*_). Figure 2C shows that this information was highly synergistic—namely that the information about the location of one mouse that can be read from the locations of the other mice can be more than double the sum of pairwise information values of that mouse with all the others: *I*(*x*_*i*_;*x*_*j*_) + *I*(*x*_*i*_;*x*_*k*_) + *I*(*x*_*i*_;*x*_*l*_). Thus, the group is not only more complex than a collection of individuals, but much more complex than even the full collection of pairs.

The remaining 75% of uncertainty about the location of each mouse is exactly the level of individuality of each mouse, which cannot be explained in terms of the location of the other mice. If there were more information about the location of one mouse from the locations of the others, then that mouse would be less ‘free’ to decide on its location. We therefore turned to characterize group behavior in terms of the combination of individual mouse traits and the dependencies between mice.

### High-order social interactions are necessary to explain group behavior

Dissecting the role of individual behavior and the dependencies between animals in shaping the group’s behavior is difficult, since we need to infer from the joint behavior what the underlying contributions of ‘pure’ mouse individuality and the nature of the interactions are. The difficulty arises since in general, for any given set of observable features of the behavior, there can be multiple models that will be consistent with these observables. We therefore used the idea of maximum entropy (ME) models from physics (Jaynes, 1957) to construct minimal models of the group, based on different order of dependencies between the animals. Since the entropy of a distribution measures its randomness of lack of structure, then among all models that are consistent with some desired feature of the data, the maximum entropy model is the most parsimonious explanation that does not make any additional assumptions beyond the required features. This minimal model is mathematically unique and can be found numerically (Schneidman et al., 2003). Such models have been successfully used to infer functional dependencies between neurons, genes, proteins and more (e.g., Schneidman et al., 2006; Ganmor et al., 2011; Lezon et al., 2007; Marre et al., 2009; Mora et al., 2010; Stephens et al., 2010; Stephens et al., 2013; Bialek et al., 2012).

We built a hierarchy of maximum entropy models to describe the group configurations, based on successive orders of correlations between the mice (one that relies only on individual behavior of the mice, one that adds pairwise dependencies between mice, third order, etc.). The relationship between these models then allowed us to dissect exactly the contribution of each order to the total group behavior.

The first-order model is one that relies only on the individual behavior of each of the mice, but assumes no dependencies among them at all. The maximum entropy model in this case is built on the observed probability of finding each mouse in one of the regions in the arena, and is exactly the independent model (that we used above), namely p^(1)^(*x*_1_, *x*_2_, *x*_3_, *x*_4_) = p(*x*_1_) p(*x*_2_) p(*x*_3_) p(*x*_4_). As it was clear already from Figure 2B, the independent model is insufficient to describe the behavior of the group.

Next, we tried to describe the group configurations using a model that takes into account both the individual behavior and the pairwise relations between mice. The minimal pairwise-based model is then given by the maximum entropy distribution that is consistent with the distribution of states that we observe for each mouse individually (i.e., first-order statistics), and the pairwise correlations between them (i.e., second-order statistics). Unlike the independent case, this cannot be performed by a simple factorized probability distribution and must be found numerically by solving an optimization problem in which we maximize the entropy with a given set of constraints. The solution of this optimization problem (see ‘Materials and methods’) is given by

$${\text{p}}^{\left(2\right)}\left(x_{1},x_{2},x_{3},x_{4}\right)=\frac{1}{Z}\exp\left(\sum_{i}\alpha_{i}\left(x_{i}\right)f_{i}\left(x_{i}\right)+\sum_{i<j}\beta_{\mathrm{ij}}\left(x_{i},x_{j}\right)f_{\mathrm{ij}}\left(x_{i},x_{j}\right)\right)\;,$$

where the parameters, *α*_*i*_(*x*_*i*_) for each mouse *i* for location *x*_*i*_ and *β*_*ij*_(*x*_*i,*_*x*_*j*_) for each pair i and j (one for every combination of locations, *x*_*i*_ and *x*_*j*_), are set such that the marginal probabilities of the model agree with the empirically observed p(*x*_*i*_) and p(*x*_*i*_,*x*_*j*_); *f*_*i*_(*x*) is an indicator function, which equals 1 when mouse i is in location *x*_*i*_ and 0 otherwise, and *f*_*ij*_(*x*_*i*_,*x*_*j*_) is an indicator function, which equals 1 when mouse i is in *x*_*i*_ and mouse j is in *x*_*j*_; Z is the normalization factor, or partition function.

We can build more complex models of group behavior by adding orders of interactions between mice. Thus, the third-order model is given by a maximum entropy distribution of a similar form, which has the same single mouse, and pairwise statistics, but also the empirically third-order statistics. This third-order model, p^(3)^, has, in addition to *α*_*i*_(*x*_*i*_)’s and *β*_*ij*_(*x*_*i,*_*x*_*j*_)’s, interaction parameters for each triplet and locations *γ*_*ijk*_(*x*_*i*_, *x*_*j*_, *x*_*k*_). The fourth-order model, p^(4)^, uses all possible correlations among mice. We emphasize that the maximum entropy models give the most parsimonious explanation of the data for each order, and therefore are not just an arbitrary ‘modeling approach’ but rather the least structured models one could build for the observed data. This hierarchy of maximum entropy models allows us to dissect the role of individual behavior, pairwise relations, triplets and so on, since every model adds a unique set of independent constraints.

**Figure 3.**
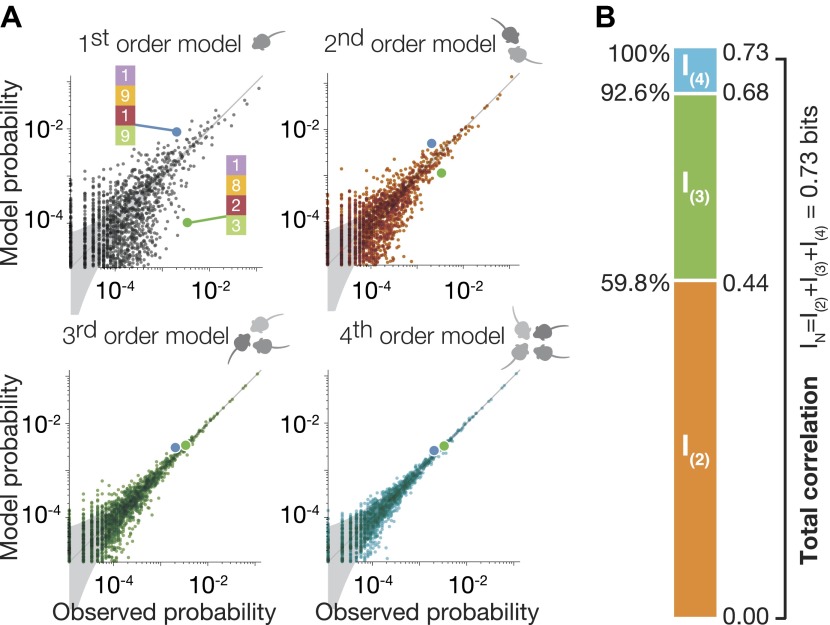
High-order maximum entropy models show the role of pairwise and triplewise interactions in shaping the group configurations of one representative group. (**A**) In each panel we present the accuracy of the corresponding ME model, from first to fourth order, in describing the empirical data for the group. Each dot corresponds to one configuration state of the group, and its probability is shown for the data (x-axis) and the prediction of the model (y-axis). The grey funnel shows the 95% confidence interval of estimation of the empirical distribution of configurations. Examples of two specific configurations are highlighted in all graphs (green and blue dot), to show improvement of model accuracy over orders. (**B**) Breakdown of the total group correlations, or multi-information *I*_*N*_, to the contribution of the pairwise interactions between mice, *I*_(2)_, triplet interactions between them, *I*_(3)_, and fourth-order contribution *I*_(4)_. **DOI:** http://dx.doi.org/10.7554/eLife.00759.007

Figure 3A shows the accuracy of the maximum entropy models of different orders in describing the empirical distribution of the spatial configurations of the mice (see ‘Materials and methods’). The top left panel shows how poorly the independent model p^(1)^ describes the empirical distribution of configurations of the group p_empirical_. This discrepancy (which was already apparent in Figure 2B) reflects the effect of the correlations among mice on the group behavior. The top right panel shows that the pairwise model p^(2)^ was a much better model of the group behavior, and captured much of these correlations in the group, but still shows considerable differences from the empirical data. Thus, the group correlations have a significant higher-order contribution. We see that p^(3)^ gave a very good approximation to the empirical data (left bottom panel), and was very close to the accuracy of p^(4)^ that relies on all orders of correlations among mice (bottom right panel). We emphasize that the comparison was performed through cross-validation, namely, ME models were fit to a randomly chosen half of the data (train data), and then compared to the empirical distribution based on the other half (test data).

To quantify the accuracy of each of these models in capturing the full group configurations, and the relative contribution of each interaction order in explaining the group behavior, we first estimated the total correlations of all orders in the group that go beyond the individual behavior of the mice. We used the ‘multi-information’ of the group (Schneidman et al., 2003), which is a generalized form of the mutual information between two variables that measures dependencies among a group of variables $I_{N}\left(\left\{x_{1},x_{2},...,x_{N}\right\}\right)$. It measures by how much the dependencies between mice change the configurations of the mice, compared to what would be expected if the mice were independent, through the difference between the entropy of the independent (first-order) model and the entropy of the empirical distribution, (see ‘Materials and methods’). *I*_*N*_ can be uniquely broken down to the sum of exact contributions of each order of correlations, when the contribution of order k to *I*_*N*_, is given by $I_{\left(k\right)}=H\left[{\text{p}}^{\left(k-1\right)}\right]-H\left[{\text{p}}^{k}\right]$, where $H\left[{\text{p}}^{k}\right]$ is the entropy of the ME models of order *k* (Schneidman et al., 2003). Here, *I*_*N*_ = *I*_(2)_ + *I*_(3)_ + *I*_(4)_, which then gives the second-order, third-order and fourth-order contributions to the correlation in the group beyond what the individuality predicts. We found that over all groups, the contribution of the pairwise ME model, given by *I*_(2)_ was 57.2% ± 10.2% of *I*_*N*_. We found that *I*_(3)_ carried nearly a third of the total correlations, and so p^(3)^ that relies on individual traits, pairwise and triple interactions between the mice explains 92.8% ± 2.9% of the correlations (Figure 3B shows as an example the results for the group shown in Figure 3A). Thus, for a group of four mice even using all pairwise interactions is not enough to capture the group behavior; the third-order interactions are therefore necessary and capture about a third of the correlation structure. This strong high-order dependency is consistent with the synergistic effect seen in Figure 2 in terms of the information that can be read about the location of a mouse from that of the other mice.

### Inferring the pairwise and triplewise functional interaction maps underlying group behavior

**Figure 4.**
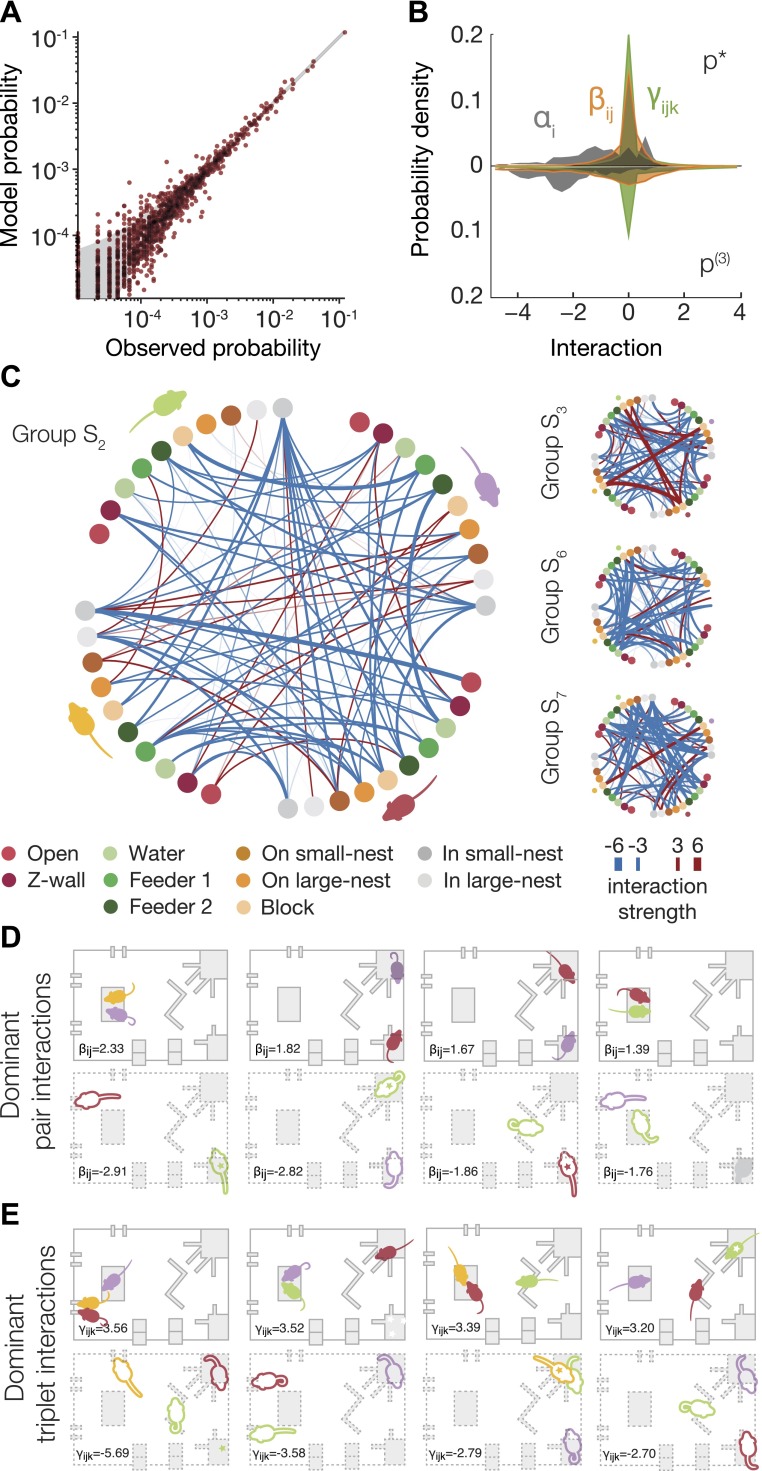
Functional social interaction maps between mice. (**A**) Accuracy of a ‘regularized’ third-order maximum entropy model of the spatial configurations of the same groups of mice from Figure 3A. Model predictions are plotted against the empirical distribution. For details of parameter selections for the regularized model see Figure 4—figure supplement 1. (**B**) The distribution of ME parameters according to the order of interactions in the regularized p^*^ model (shown above the horizontal line), compared to the model without regularization (shown below the line). The distribution is over parameters of all eight groups of SE mice taken together. (**C**) Full pairwise interaction maps for four representative groups. (Group S2 is magnified as it is used in following panels.) In each of these maps, the colored dots represent the location of a mouse according to the color coding in the bottom of the figure. The colors of the mice are depicted near their corresponding locations. The color of a vertex shows whether the interaction is positive (red) or negative (blue) and its width reflects the interaction strength. An alternative presentation of all the pairwise interaction parameters is shown in Figure 4—figure supplement 2. (**D**) The dominant positive and negative pairwise interactions are shown overlaid on a diagram of the arena. ‘Filled mice’ show positive interactions, and ‘empty mice’ show negative interactions. A star denotes that the mouse is on the nest. The value of the corresponding interaction is shown on the bottom of each panel. (**E**) The dominant positive and negative triplewise interactions for the same group as in **D**, overlaid on a diagram of the arena. **DOI:** http://dx.doi.org/10.7554/eLife.00759.008

The maximum entropy models we have used to describe the group behavior are a generalized form of the Potts model from statistical physics, which describes the behavior of spins in a lattice in terms of the interactions between them (Landau and Lifshitz, 1980). We therefore interpreted the parameters of our ME models as the interactions between the mice at the different locations; these reflect functional (rather than physical) dependencies between the animals. To give as compact an explanation of the social interaction between animals as possible (and to avoid overinterpretation of the parameters we found in the ME model), we tried to identify the dominant and irreducible dependencies between animals. We therefore constructed a third-order ME model, where we tried to minimize the number of parameters of the model. Specifically, we fitted the maximum entropy model but added a constraint in the form of a cost for every interaction term that is not zero (see ‘Materials and methods’ and Figure 4—figure supplement 1). This standard regularization approach gives a model, p^*^, that is nearly as accurate as the full third model (Figure 4A), but uses far less parameters (Figure 4B and Figure 4—figure supplement 1). Figure 4C shows all the pairwise interactions between the mice for one of the groups (Figure 4—figure supplement 2). We found that most pairwise parameters (or functional interactions) were negative, making the corresponding configurations less common than predicted from single mouse preferences, and positive interactions were less common and weaker, making the corresponding configurations occur more than expected from single mouse traits. Figure 4D,E show the most dominant pairwise and triplewise interactions for one group, respectively, overlaid on a drawing of the arena. Importantly, the interaction maps show that mere physical limitations do not play a key factor in shaping the group configurations. In particular, we did not find strong high-order interactions for configurations in which more than two mice are in the same location. (i.e., there is no exclusion of these configurations that requires a special interaction that would ‘prevent’ this from happening).

**Figure 4—figure supplement 1.**
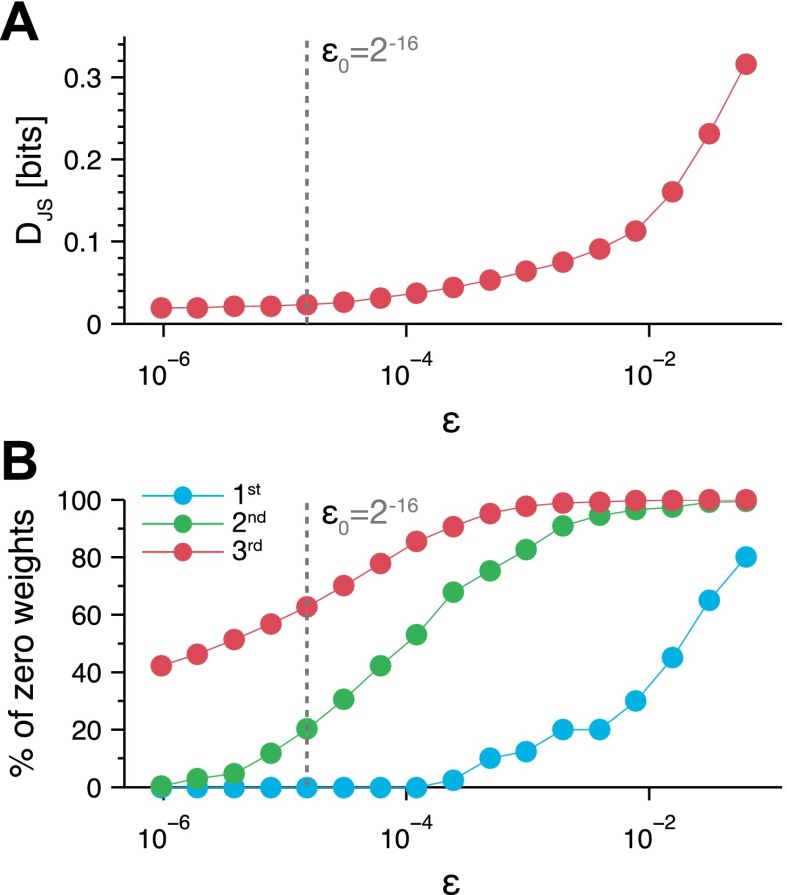
Tradeoff between generalization and accuracy of the maximum entropy model. We found the 3^rd^ order maximum entropy model for the mice configurations, with an additional penalty term that minimized the number of non-zero parameters (see Materials and methods). The balance between maximizing the model's entropy and minimizing the penalty is controlled by parameter *∈*. (**A**) The effect of the tradeoff parameter on the accuracy of the model is shown as the Jensen–Shannon divergence (DJS) between the third order maximum entropy model with the penalty term and the model without the penalty term (as in Figure 3). The Jensen–Shannon divergence equals 0 when the two models are identical, and would be 1 at its maximal value—when the two distributions are distinct. The results are from the second day of the same group as in Figure 3a. (**B**) The fraction of parameters that equal zero is shown for each order (1^st^, 2^nd^ and 3^rd^ order parameters of the maximum entropy model) is shown as a function of *∈*. The chosen value $\in_{0}=2^{-16}$, which we used in Figure 4 is marked by a dashed line on the graphs. **DOI:** http://dx.doi.org/10.7554/eLife.00759.009

**Figure 4—figure supplement 2.**
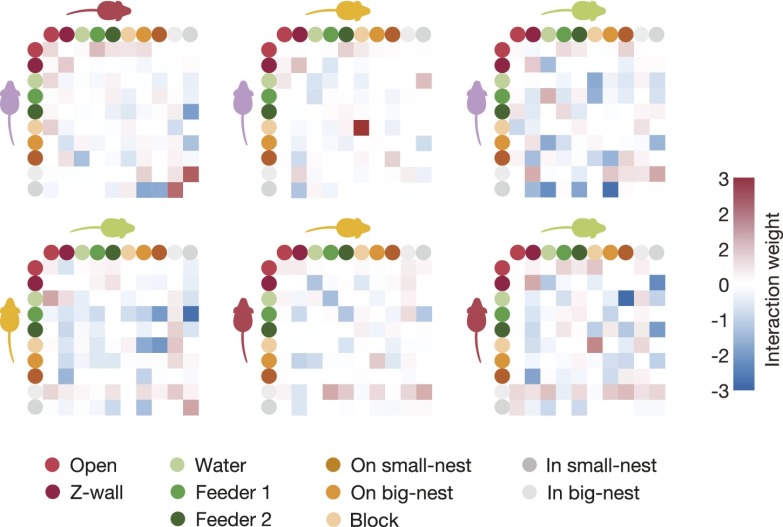
All pairwise interactions of a typical group. These interactions are the weights of the second-order interactions in the regularized third-order maximum entropy model. Each panel corresponds to one pair of mice, and its rows and columns correspond to the locations within the arena according to the legend at the bottom of the figure. **DOI:** http://dx.doi.org/10.7554/eLife.00759.010

### Social interactions and correlated group behavior depend on past environment

**Figure 5.**
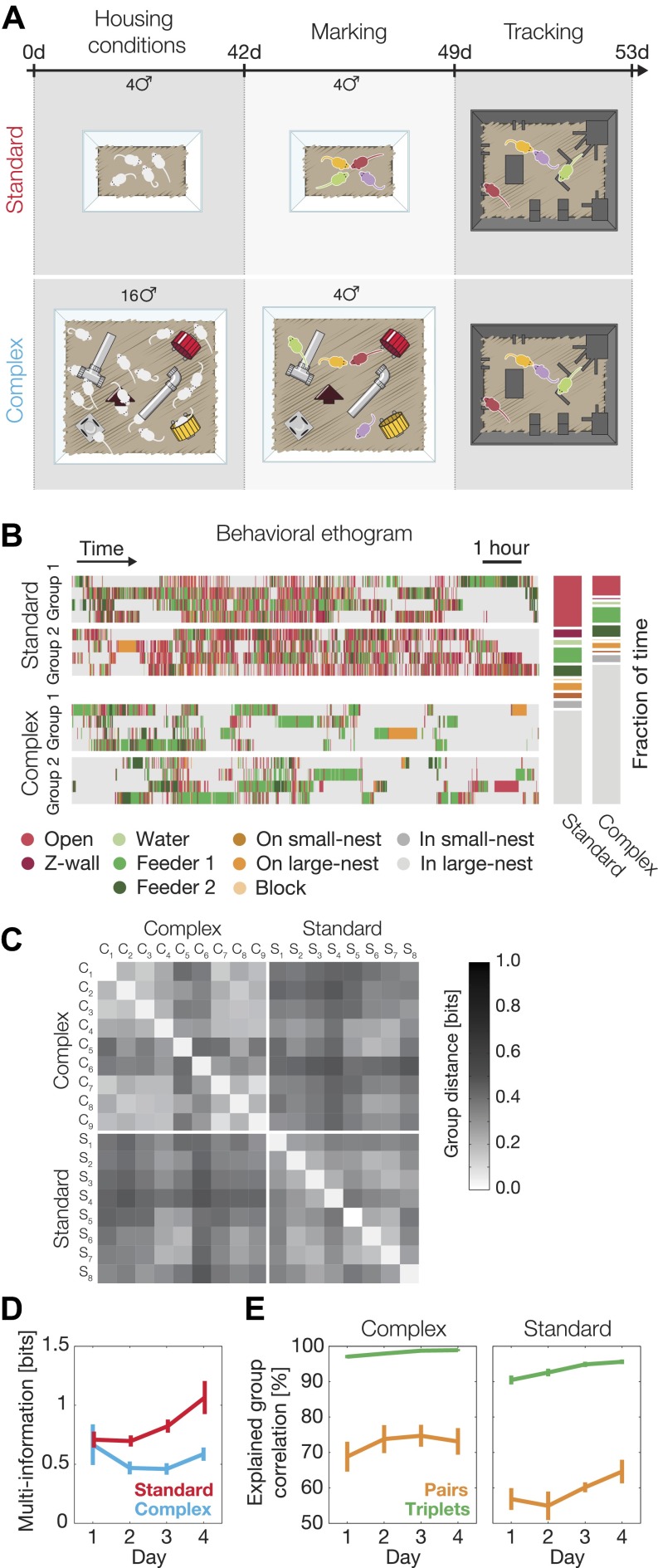
Environmental background changes group behavior and interactions. (**A**) Experimental design. At the age of 4 weeks (day 0 of the experiment), mice were separated into two different housing conditions: standard environment and complex environment. After 6 weeks, groups of four mice from both treatments were color marked, returned to their cages for an additional week, and then put in the arena for recording their group social behavior. (**B**) Behavioral ethograms of two representative groups from each treatment (left). Data shown in these panels is for the 12 hr of the second day. Average percentage of time spent at the different regions over all groups for each treatment (right). (**C**) Similarity of group behavior between all SE and CE groups. For each pair of groups, the Jensen–Shannon divergence between the third-order maximum entropy models of the groups was calculated. Matrix entries are ordered by their corresponding SE or CE label. (**D**) Total group correlation (multi-information, *I*_*N*_) of the SE and the CE groups over 4 consecutive days. (**E**) The contribution of each order of interaction to the total correlation in the groups. Figure 5—figure supplement 1 presents the differences in the distribution of the fraction of information about the location of one mouse that can be ‘read’ from the location of the other mice for SE and CE groups. **DOI:** http://dx.doi.org/10.7554/eLife.00759.011

Since the nature of the environment and availability of resources determine population density, aggression, dominance, and territoriality in mice (Bronson, 1979; Haemisch et al., 1994; Van Loo et al., 2001), we asked how raising mice in a complex and more populated environment (Sztainberg and Chen, 2010; Sztainberg et al., 2010) might affect their group behavior. We found that groups raised in standard laboratory conditions environment (SE, n = 8) and those raised in a complex environment (CE, n = 9; Figure 5A), already showed distinct behavior at the individual level, as CE mice spent significantly more time inside the large nest and less time outside (Figure 5B). But more importantly, we found clear differences between SE groups and CE ones in terms of the overall group behavior and, in particular, the nature of the interactions in the group that go beyond single mouse individuality. Given their accuracy in describing the group behavior, we used the third-order models that we fitted for each group separately to compare the distribution of the spatial configurations in the arena. We found that CE groups were more similar to other CE groups than to SE groups (and vice versa) in terms of the overall distribution of observed configurations of the mice (Figure 5C).

**Figure 5—figure supplement 1.**
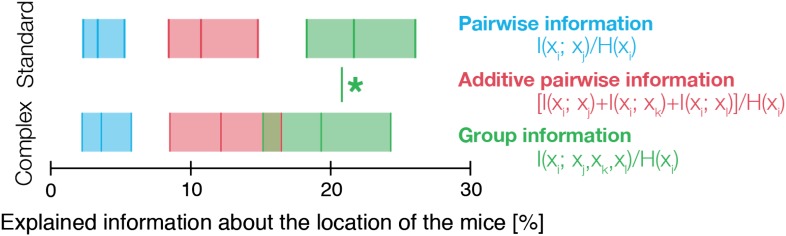
Histogram of the fraction of information about the location of one mouse that can be ‘read’ from the location of the other mice. Top: mice in standard conditions. We estimated pairwise mutual information between all pairs, and normalized by mouse entropy (main text). Histogram of values over all mice is shown as a horizontal bar in blue, with median as central line, and the left and blue boxes show the range of 25% and 75% values, correspondingly. The histogram of the fraction of information that can be read from the joint location of the other three mice is shown in green. The sum of pairwise information for each mouse with the others is shown in red. Bottom: same as top, but for complex environment groups. Green star denotes the significant difference between the histograms of the group information values of complex and standard groups (Klomogorov–Smirnoff, p<0.0001). **DOI:** http://dx.doi.org/10.7554/eLife.00759.012

We found that the total group correlation *I*_*N*_ values of the SE and CE groups were similar on the first day, but then the SE groups became more correlated. In contrast, the correlations among the CE mice remained unchanged (Figure 5D). In other words, there was a progressive increase in social correlation, or ‘socialization’ in the SE groups, which was absent in the CE groups. We emphasize that these are differences at the level of group behavior that go beyond the differences between the individual (single animal) behavior patterns of SE and CE mice.

Finally, we found that the contribution of the different orders of interactions to the group behavior differed significantly between the SE and CE groups. In the CE groups the contribution of pairwise interaction to the full group correlations was higher than in SE groups (74.6% ± 2.5% in CE groups compared to 61.9% ± 2.4% for the SE ones, averaged over all four days). The dominance of the low-order interactions was also reflected by the virtual lack of contribution of fourth-order interaction in the CE groups, whereas the SE groups showed more complex high-order structure (fourth-order interactions contribution to the total correlation in the SE groups was 6.0% ± 0.7% [Figure 5E]). The larger role of pairwise interactions in shaping the group’s behavior is also reflected in the information about the location of one mouse that can be read from the locations of the other three mice. In the CE groups the information from three mice about the fourth was far less synergistic than in the case of SE groups (Figure 5—figure supplement 1). Thus, mice exposed to a complex environment during adolescence were more individual, and their weaker group correlations relied more on pairwise rather than higher-order dependencies.

## Discussion

Quantifying social interactions presents an ethological challenge both experimentally and theoretically (Adolphs, 2010). While even solitary species display social behavior such as mating, aggression, and maternal care, species that live in groups display profoundly more complex social repertoires (Silk, 2013). This group behavior, ideally dissected into individual and group parts, is also likely to depend on the environmental context (Insel and Fernald, 2004). Thus, understanding group social behavior requires a framework combining an experimental system for recording group behavior with high resolution both spatially and temporally in a reliable manner over long time windows, and a mathematical formalism to quantify the nature of interactions and their contribution to the group’s behavior.

Mice are an ideal model organism for investigating social behavior in mammals and the neural mechanisms that underlie it. Together with the ability to manipulate their genomic make-up (Lewandoski, 2001), and record neural activity (electrophysiologically or optically), mice live in groups and form diverse societies with different characteristics such as group size, hierarchy, aggression, and social tolerance (Bronson, 1979). However, despite the complex nature of their social organization, the current methodologies used for analysis of social behavior in mice have focused mainly on dyadic interactions such as in the classical three-chamber social approach test and the partition test (Silverman et al., 2010). One common approach has been to record, via video, the interaction between two animals and then have defined behaviors scored by trained human observers (Moretti et al., 2005). This allows for identifying intricate social behaviors and can provide new insights about underlying features of social interaction, but demands immense human resources and is prone to human error. Another approach has focused on the behavior of one individual towards other restricted conspecifics (Nadler et al., 2004; Moy et al., 2008; Ben-Ami Bartal et al., 2011), which allows for an automated behavioral scoring system. However, since only one animal is free to roam, its behavior might be altered due to the synthetic dynamics of interactions. de Chaumont et al. (de Chaumont et al., 2012) reported an automated video tracking system for social interaction between two rats, which were analyzed based on their relative locations in a 10 min test. This method holds the advantages of both rats roaming free and the use of an automated system; however, the ability to distinguish between different behaviors is limited.

The system we introduce here enables automatic tracking of group behavior of mice in the dark, over long periods of time, and in a semi-natural environment, with high spatiotemporal resolution while maintaining individual identities. Similar systems for tracking multiple individuals (Freund et al., 2013) allow for tracking animals over long periods of time, using radio-frequency identification (RFID) tagging of individual animals. We note that RFID-based systems allow for the tracking of a very large number of animals, whereas our tracking capacity depends on the number of distinguishable dyes and spatial marking patterns on the mice (our preliminary results suggest we can expand even the current system to more than 10 mice). However, the strength of our system is in its much higher spatial and temporal resolution, and the ability to track and analyze details of individual behavior of the animals and between them.

Clearly, these kinds of systems would change the way individual and social behavior can be studied and quantified. The recent work by Freund et al. used the tracking of many mice over several months to study the individual mouse behavior within a large group and showed correlation between the roaming behavior of a mouse and the level of neurogenesis in its hippocampus (Freund et al., 2013). The work we have presented here addresses the complementary question of the nature of group behavior that goes beyond individual traits, focusing on the interactions between animals. Our results show the limitations of individual-based and even pairwise-based approaches, and identify irreducible high-order structure among mice. Moreover, although every individual group is likely to have its own unique nature, hierarchy, traits and rules, we were still able to identify universal features of the groups that govern their behavior and distinguish different behavioral contexts. Combining detailed behavioral and genetic analysis at the level of individuals as seen in Freund et al. (2013), in association with the kind of group analysis used, may enable the identification of genetic and neuronal correlates of complex social interactions.

Our analysis of the groups relied on a representation of the mice in their preferred locations in the arena. This is a discretized version of the full physical space, but even at this level the number of potential group configurations, which is exponential in the number of animals, is very large. We found strongly correlated group structure among the animals, which dictate which configurations are permitted and which are not. Moreover, we found that more information was obtained from the joint position of the other mice than from summing all the information provided by the interactions between the pairs of mice. To assess the contribution of individuality and of pairwise and higher-order interactions among the mice, we used tools from information theory to quantify any kind of dependency, of any order, be it linear or non-linear. Intriguingly, the pairwise-based model of the group that assumed no higher-order contribution could only explain approximately 60% of the correlation structure in the group, whereas models that included also third-order dependencies (but not fourth-order ones) captured approximately 90% of the group correlation structure. How should one interpret these results together? The ME model shows how well we can describe the distribution, whereas the information about location reflects how deterministic the behavior of one mouse is given the others. What we can read about the location of a mouse from the location of the others is much more than what one would naively expect from the pairwise relations between mice, which amounts to approximately 5%. This strong synergistic effect is the result of high-order dependency between the animals (which the maximum entropy models reflect), but it is still the case that three-quarters of the uncertainty we have about the location of a mouse comes from its own individuality. That is, the mice still have a significant individual component, even given the other mice.

The need for models that include high-order interactions is surprising, since intuitively one might have expected that it would be possible to construct a mathematically accurate description of the group once all the interactions between pairs of mice were known. After all, most social behaviors, such as fights, chases, courtship, and grooming, are usually observed in pairs. Our analysis of the social interaction network underlying the group behavior relied on a family of maximum entropy models, which enabled us to uniquely dissect the contribution of different orders of correlations in the group. This approach has been useful for different biological networks, from small to large networks of neurons (Schneidman et al., 2006; Shlens et al., 2006; Marre et al., 2009; Ohiorhenuan et al., 2010; Ganmor et al., 2011), genes (Lezon et al., 2007), T cells (Mora et al., 2010), letters in words (Stephens and Bialek, 2010), the structure of images (Stephens et al., 2013), and birds in large flocks (Bialek et al., 2012). Interestingly, in almost all these cases the contribution of pairwise interactions was very large and dominated the network structure, especially in small networks—in clear contrast to what we found for the mice.

The parameters of the maximum entropy models can be interpreted as functional social interactions between the animals (similar to the parameters of the corresponding Potts models from physics). We emphasize that these functional interactions reflect statistical dependencies, and will probably differ from explicit physical interactions between the animals that one could measure. Yet, these statistical dependencies highlight the most prominent relations that underlie the patterns of group behavior. The strongest functional interactions corresponded not to the most frequent events, but rather to those events that are most surprising or not predicted from lower orders of interactions, thus presenting interactions of a truly social nature. Our results show that the dominant interactions in the group are negative ones, namely compound configurations that tend not to happen compared to expectations based on individual behavior. This may suggest competition as a dominant force in the social structure. In addition, the relative sparseness of the interaction maps indicates that even a small number of social events can have a strong, macroscopic impact on the group.

As an example of how the combination of group tracking and the analysis based on information theory tools can enhance our understanding of the effect of external factors on group behavior, we compared the effects of different environmental exposures on social behavior. We found that growing up in a complex environment with more mice, which better resembles a natural habitat, resulted in groups that were far less correlated as a group, and their social structure could be explained to a much larger degree based on pairwise interactions. We suggest that this approach could now enable the quantitative characterization of many different aspects of group behavior that have so far only been studied in much more restricted set-ups, such as the effects of stress, rewards, and learning on the group.

Several technical and mathematical issues should be further explored to allow the extension of our approach to other groups of animals and contexts. First, we reiterate that our analysis has focused on a reduced description of the mice configurations (regions of interest), and not absolute or relative coordinate space. While it is not immediately clear how to construct such models, they have the potential to reveal new features and dependencies in the group, and with respect to cues from the environment as well. Moreover, it would be interesting to consider how our analysis might be related to more standard hierarchy models in groups. Second, we have focused on the joint configurations of the mice at given time points, and have not included temporal correlations between them. Third, it will be interesting to consider how the number of animals in the group affects the nature of group correlations and the contributions of the different interaction orders. Preliminary results suggest that our system can be expanded in terms of tracking more mice using additional dyes and using spatial color patterns on the mice.

We expect that mapping of the social interactions among other and larger groups of animals, and their dynamics, will change our understanding of group behavior in terms of the interplay between genetics, individuality, environment, and social hierarchy. Of particular interest would be the extension of our approach to study animal models of maladaptive social behavior. For example, our analysis would allow identifying mutants that rely on different kinds of low or high-order interactions compared to wild type littermates; such analysis would be useful for studying mechanisms underlying social intolerance and group stability, as well as models of autism and schizophrenia. Because our approach is based on high throughput as well as high spatiotemporal resolution, it may also be useful in detecting subtle changes in social behavior in mice that may not be detectable in standard social behavior paradigms even for standard parameters such as exploration, feeding or locomotor activity.

## Materials and methods

### Arena

Mice were studied in a specialized arena designed for automated tracking of individual and group behavior. The arena consisted of an open 70 cm × 50 cm × 50 cm box and included the following objects: Z-shaped wall, a water dispenser, two feeders, a small nest and a large nest, an elevated block, and two elevated ramps (Figure 1—figure supplement 1). Food and water were given ad libitum. Two UVA fluorescent lamps (18 W) were placed 3 m above the arena’s floor to illuminate the surrounding area with 370–380 nm ‘black light’. To avoid reflections from white objects in the room, a black curtain was drawn from the fluorescent lamps down to the arena. A color sensitive camera (Panasonic Color CCTV, WV-CL924AE) was placed 1 m above the arena. The camera analog input is converted to digital information with a digitizer (Picolo Diligent frame grabber board), and recoded on a standard computer. Mice trajectories were automatically detected offline using specially written software in Matlab (Mathworks, Natick, MA).

### Animals

Adult male ICR mice (Harlan Laboratories, Jerusalem, Israel) were used for the standard and complex environment experiment. Throughout the experiments, the animals were maintained in a temperature-controlled mouse facility (22°C ± 1) on a reverse 12 hr light–dark cycle. Food and water were given ad libitum. All experimental protocols were approved by the Institutional Animal Care and Use Committee of The Weizmann Institute of Science.

### Color marking

Mice were mildly anesthetized with a mix of ketamine (70 mg/kg) and xylazin (7 mg/kg). Their eyes were protected against drying using eye gel (viscotears liquid gel; Alcon). The fur of the mice was stained using a regular brush with fluorescent semi-permanent hair dyes that glow under black light. The fur was dried with a fan (low power and heat) for 3 min. After awakening, mice were kept in separate carton boxes for 4 hr before reunion. Mice were introduced to the arena for tracking 5 days after the fur staining. The dyes used were Electric banana, (HCR 11012), composed of natural ingredients, Virgin snow white (HCR 11033), and Raven (HCR 11007), from Tish & Snooky’s (manicpanic.com), and High octane orange, from specialeffectsusa.com.

**Table:** 

Color under black light	Color under regular light	Dyes ratio
Green	Yellow	100% Electric banana
Purple	White	100% Virgin snow white
White	Yellowish	20% Electric banana, 80% Virgin snow white
Red	Red	80% High octane orange, 20% Electric banana
Orange	Orange	20% High octane orange, 80% Electric banana
Black	Black	100% Raven

### Standard and complex environment conditions

At the age of weaning (4 weeks), mice were randomly distributed into 2 types of groups: standard environment (SE) mice that were housed in groups of 4 in standard laboratory cages, and complex environment (CE) mice that were housed in groups of 16 male mice in a relatively spacious and complex cage, with a variety of objects such as shelters, tunnels, running wheels, and mouse nest boxes (Sztainberg and Chen, 2010). After a period of 6 weeks, CE mice were randomly divided into groups of four, color marked, and introduced to the novel arena, as the SE mice, for analysis of group social behavior.

### Mouse tracking

Mice were identified and tracked automatically, according to their fur colors, which were learned from labeled data. Because of low signal-to-noise ratio, due to the dim lighting and the camera’s sensitivity, some frames had reflection artifacts or missing parts. To overcome this noise, we used a Bayesian model to infer the most likely location of a mouse given the observed location of connected colored blobs. Validation of the tracking algorithm was performed by comparing the algorithm’s performance to human labeling of 500 randomly chosen frames, which gave 99.6% accuracy.

### Sampling rate

The raw camera acquisition rate was 25 frames/s (40 ms per frame). In analyzing the configurations of the mice we used a lower resolution of 240 ms per frame, as this did not have a major effect on the state distribution, but was more robust to single frame noise.

### Estimating mutual information about mouse locations

The uncertainty about the location of mouse *i* is given by the entropy of its location distribution, $H\left(x_{i}\right)=-\sum_{x_{i}}\text{p}\left(x_{i}\right)\log_{2}\text{p}\left(x_{i}\right)$. The mutual information between the location of mouse *i* and that of mouse *j* is given by $I\left(x_{i};x_{j}\right)=H\left(x_{i}\right)-H\left(x_{i}|x_{j}\right)$, where $H\left(x_{i}|x_{j}\right)=-\sum_{x_{i},x_{j}}\text{p}\left(x_{i},x_{j}\right)\log_{2}\text{p}\left(x_{i}|x_{j}\right)$ is the average conditional entropy or uncertainty about mouse *i* given the location of mouse *j*. The fraction of the uncertainty about the location of mouse *i* that can be extracted from the location of mouse *j* is then given by $\frac{I\left(x_{i};x_{j}\right)}{H\left(x_{i}\right)}$. The fraction of uncertainty about the location of mouse *i* that can be ‘read’ from the joint location of the three other mice is given by

(1) $$\frac{I\left(x_{i};\left\{x_{j},x_{k},x_{l}\right\}\right)}{H\left(x_{i}\right)}$$

The naive additive pairwise information fraction was defined as

(2) $$\frac{I\left(x_{i};x_{j}\right)}{H\left(x_{i}\right)}+\frac{I\left(x_{i};x_{k}\right)}{H\left(x_{i}\right)}+\frac{I\left(x_{i};x_{l}\right)}{H\left(x_{i}\right)}$$

### Group correlations and multi-information

The total correlation of all orders between mice was quantified by the multi-information of the group (Schneidman et al., 2003)

(3) $$I_{N}\left(\left\{x_{i}\right\}\right)=\sum_{j}H\left(x_{j}\right)-H\left(\left\{x_{i}\right\}\right)=\sum_{\left\{x_{i}\right\}}\text{p}\left(\left\{x_{i}\right\}\right)\log_{2}\frac{\text{p}\left(\left\{x_{i}\right\}\right)}{\prod_{j}\text{p}\left(x_{j}\right)}$$

where the joint entropy of the mice configurations is defined by

(4) $$H\left(\left\{x_{i}\right\}\right)=-\sum_{\left\{x_{i}\right\}}\text{p}\left(\left\{x_{i}\right\}\right)\log_{2}\text{p}\left(\left\{x_{i}\right\}\right)$$

(where {*x*_*i*_}={*x*_*1*_,*x*_*2*_,*x*_*3*_,*x*_*4*_}) and the entropy of the independent mice model or the sum of the entropies of the mice is $\sum_{i}H\left(x_{i}\right)$.

### Maximum entropy models for mice configurations

For a given set of observed average functions of the group, $<f_{i}\left(\left\{x_{i}\right\}\right)>$, the maximum entropy model, which is the minimally structured model that is consistent with these measured functions, is given by

(5) $$\text{p}\left(\left\{x_{i}\right\}\right)=\frac{1}{Z}\exp\left(\sum_{i}\lambda_{i}f_{i}\left(\left\{x_{i}\right\}\right)\right)$$

where *λ*_*i*_ are set such that the average of each *f*_*i*_ under the model,<*f*_*i*_>_p_ is identical to the empirical expectation value, <*f*_*i*_>_empirical_, and *Z* is the normalization or partition function (Jaynes, 1957).

For each group of mice, we then find a hierarchy of maximum entropy models that gives the minimal description of the mice configurations, relying only on pairwise correlations between mice (p^(2)^), pairwise and triplewise correlations (p^(3)^), and all correlations (p^(4)^; pairs, triplets, and quadruplet correlations). The constraints of each of these maximum entropy models are the empirical marginal of different orders, that is, single mice p_empirical_ (*x*_*i*_), pairs p_empirical_ (*x*_*i*_,*x*_*j*_), and so on. For example, the pairwise model is the maximum entropy distribution over all mice, such that the marginal probabilities p(*x*_*i*_) and p(*x*_*i*_,*x*_*j*_), the pairwise marginal probability to find mouse *i* and mouse *j* in location *x*_*i*_ and *x*_*j*_, are the same as empirically found in the data. Formally, we seek $p\left(\left\{x_{i}\right\}\right)$ that maximizes

(6) $$\begin{array}{l} L\left(\text{p}\left(\left\{x_{i}\right\}\right),\left\{\alpha_{i}\left(x_{i}\right)\right\},\left\{\beta_{ij}\left(x_{i},x_{j}\right)\right\}\right)=-\sum_{\left\{x_{i}\right\}}\text{p}\left(\left\{x_{i}\right\}\right)\log_{2}\text{p}\left(\left\{x_{i}\right\}\right)\\ -\sum_{i}\sum_{x_{i}}\alpha_{i}\left(x_{i}\right)\left(\text{p}\left(x_{i}\right)-{\text{p}}_{\text{empirical}}\left(x_{i}\right)\right)\\ -\sum_{i<j}\sum_{x_{i},x_{j}}\beta_{ij}\left(x_{i},x_{j}\right)\left(\text{p}\left(x_{i},x_{j}\right)-{\text{p}}_{\text{empirical}}\left(x_{i},x_{j}\right)\right)\\ -\lambda_{0}\left(\sum_{\left\{x_{i}\right\}}\text{p}\left(\left\{x_{i}\right\}\right)-1\right) \end{array}$$

The resulting maximum entropy distribution is given by

(7) $${\text{p}}^{\left(2\right)}\left(\left\{x_{i}\right\}\right)=\frac{1}{Z_{2}}\exp\left(\sum_{i}\sum_{x_{i}}\alpha_{i}\left(x_{i}\right)f_{i}\left(x_{i}\right)+\sum_{i<j}\sum_{x_{i},x_{j}}\beta_{ij}\left(x_{i},x_{j}\right)f_{ij}\left(x_{i},x_{j}\right)\right)$$

where the Lagrange multipliers *α*_*i*_(*x*_*i*_) and *β*_*ij*_(*x*_*i*_,*x*_*j*_) have to be chosen to satisfy the constraints, and *f*_*i*_(*x*_*i*_) is an indicator function which equals 1 if mouse *i* is in location *x*_i_, and 0 otherwise; the partition function Z_2_ is a normalization factor.

The maximum entropy models were fit using a combination of the generalized iterative scaling algorithm (Darroch and Ratcliff, 1972), and a maximum-likelihood optimization using a variant of the gradient descent algorithm with line search (Nesterov, 2005).

The maximum entropy models of different orders form a hierarchy of correlation-based descriptions of the mice, from p^(1)^ where all mice are independent, to p^(4)^ which is an a description that allows arbitrary complex interactions; their entropies decrease monotonically toward the true entropy.

(8) $$H\left[{\text{p}}^{\left(1\right)}\right]\geq H\left[{\text{p}}^{\left(2\right)}\right]\geq \ldots \geq H\left[{\text{p}}^{\left(\text{N}\right)}\right]=H$$

The multi-information *I*_*N*_ = *H*[p^(1)^] − *H*[p^(N)^] can be broken down to the sum of contributions of each order of correlation, where the *k*’th order contribution is given by *I*_(*k*)_ = *H*[p^(k-1)^] − *H*[p^(k)^], and *I*_*N*_ = *I*_(*2*)_ + *I*_(*3*)_ + … + *I*_(*N*)_.

### Regularized maximum entropy models

To build a more compact model for the mice configurations and isolate the significant functional correlations between the mice, we constructed a model, p^*^, for the mice configurations that has the maximal entropy given a set of constraints, but also minimizing the total sum of the non-zero parameters of the model. Thus we added a penalty term (‘regularization’), to the standard maximum entropy optimization problem from equation 6, and maximize

(9) $$L\left(\text{p}\left(\left\{x_{i}\right\}\right),\left\{\alpha_{i}\left(x_{i}\right)\right\},\left\{\beta_{ij}\left(x_{i},x_{j}\right)\right\},\left\{\gamma_{ijk}\left(x_{i},x_{j}\right)\right\}\right)-\varepsilon_{0}\left(\sum_{i}\left|\alpha_{i}\right|+\sum_{i<j}\left|\beta_{ij}\right|+\sum_{i<j<k}\left|\gamma_{ijk}\right|\right)$$

where $\varepsilon_{0}$ is an adjustable parameter that controls the trade off between maximizing the entropy and minimizing the total sum of absolute values of the parameters or the *L*_1_ norm, also known as lasso regularization (Dudık et al., 2007).

### Measuring similarity between group configurations

Similarity between groups was quantified by the Jensen–Shannon divergence (D_*JS*_) between the regularized third-order models of the groups (Lin, 1991). Since the mice were arbitrarily labeled, we used the permutation of mouse identities that gave the smallest value of D_*JS*_ between two groups. Thus the distance between groups *i* and *j* is

(10) $$d\left(i,j\right)=\underset{\pi }{\min}D_{js}\left({\text{p}}_{i}\left(\left\{x_{k}\right\}\right),{\text{p}}_{j}\left(\pi \left\{x_{k}\right\}\right)\right)\;,$$

where *π* is a permutation of the mice labels such that

(11) $$\pi \left(x_{1},x_{2},x_{3},x_{4}\right)=\left(x_{k_{1}},x_{k_{2}},x_{k_{3}},x_{k_{4}}\right)$$

where $k_{1}\in \left\{1,...,4\right\}$ and are unique.
