# Correction: High-order social interactions in groups of mice

**DOI:** 10.7554/eLife.03602

**Published:** 2014-06-11

**Authors:** Yair Shemesh, Yehezkel Sztainberg, Oren Forkosh, Tamar Shlapobersky, Alon Chen, Elad Schneidman

Shemesh Y, Sztainberg Y, Forkosh O, Shlapobersky T, Chen A, Schneidman E. 2013. High-order social interactions in groups of mice. *eLife* **2**:e00759. doi: http://dx.doi.org/10.7554/eLife.00759. Published September 3, 2013

We incorrectly stated that mice do not see UV light. We would therefore like to correct the following sentence in the ‘Materials and methods’, ‘Two UVA fluorescent lamps (18 W) were placed 3 m above the arena's floor to illuminate the surrounding area with 370–380 nm ‘black light’ (which the mice cannot see)’, to read ‘Two UVA fluorescent lamps (18 W) were placed 3 m above the arena's floor to illuminate the surrounding area with 370–380 nm ‘black light’’.

The same erroneous statement appeared in the legend of Figure 1—figure supplement 1 and should be corrected there as well.

This mistake has no bearing on the behavioral results that we presented.

The article has been corrected accordingly.

